# Gossypiboma discovered 24 years after prostate surgery, a forgotten but never forgiven complication

**DOI:** 10.1093/jscr/rjac464

**Published:** 2022-10-11

**Authors:** Gabriel A Molina, Germanico Fuentes, Andres Jimenez, Estefany J Proaño, Paulina E Chango, Maria Isabel Uzcategui, Ronald S Alvear, Cristina B Rubio

**Affiliations:** Department of General Surgery, Hospital Metropolitano & Universidad San Francisco de Quito (USFQ), Quito, Ecuador; Department of General Surgery, Iess Quito-Sur, Quito, Ecuador; Department of General Surgery, Iess Quito-Sur, Quito, Ecuador; Department of General Surgery, Iess Quito-Sur, Quito, Ecuador; Department of Occupational Health and Safety, Iess Quito-Sur, Quito, Ecuador; Department of General Surgery, Iess Quito-Sur, Quito, Ecuador; Ministry of Health, Rural Medicine, Quito, Ecuador; Universidad San Francisco (USFQ), School of Medicine, Quito, Ecuador

**Keywords:** Gossypiboma, Surgical Complications, Surgery

## Abstract

Forgetting gauze or “a surgical drape” inside a patient after surgery is a rare medical error. It can lead to severe complications, high hospital costs and medico-legal implications. As a result, this complication is often not reported, mainly to avoid retaliation and because it can initiate extensive critical press coverage. This technical oversight may be just the tip of an iceberg concerning the reality of surgical errors; therefore, the entire surgical team must focus on prevention, continuing medical education and strict adherence to protocols and counting guidelines to minimize their incidence. We present the case of a 76-year-old patient with an acute abdomen; after an initial evaluation, a gossypiboma was discovered, which was forgotten 24 years after prostatectomy.

## INTRODUCTION

Postoperative complications in surgery are sometimes unavoidable; however, some complications are the result of human error [1, 2]. For example, gossypiboma occurs when a gauze or surgical swab is forgotten after surgery [3]. It can result in severe morbidity and even mortality. As it is never foreseen, it is often misdiagnosed and not reported, yet when it does happen, the patient and the surgeon will never forget it [3, 4].

We present a case of a 76-year-old patient in which gossypiboma was discovered 24 years after prostatectomy.

## CASE REPORT

The patient is an otherwise healthy 76-year-old patient with a past medical history of hypertension and a laparotomy due to a prostatectomy 24 years ago for benign prostatic hyperplasia. He presented to the emergency room with a 12-hour history of acute abdominal pain; the pain began in the lower abdomen; at first, it was mild and was accompanied by nausea; nonetheless, as time passed, the pain became unbearable and was followed by severe episodes of vomits. Therefore, he was brought immediately by his family.

On clinical examination, the patient was found to be tachycardic and dehydrated; he had severe pain on palpation in the lower abdomen with tenderness, and his vital signs at that time were: body temperature: 37°C, heart rate: 112 beats × min, blood pressure: 100/50, respiratory rate: 21 × min, and SpO2: 97%.

Surgical consultation was required, and complementary exams were requested, revealing leukocytosis: 18 000/μl, neutrophilia: 9500/μl, an elevated C-reactive protein: 25 mg/dl, a high creatinine: 2.6 mg/dl and a slight hyperkalemia 5.8 mmol/l.

Appendicitis was among the differential; however, a contrast-enhanced abdominal computed tomography (CT) was requested due to his pain.

**Figure 1:**
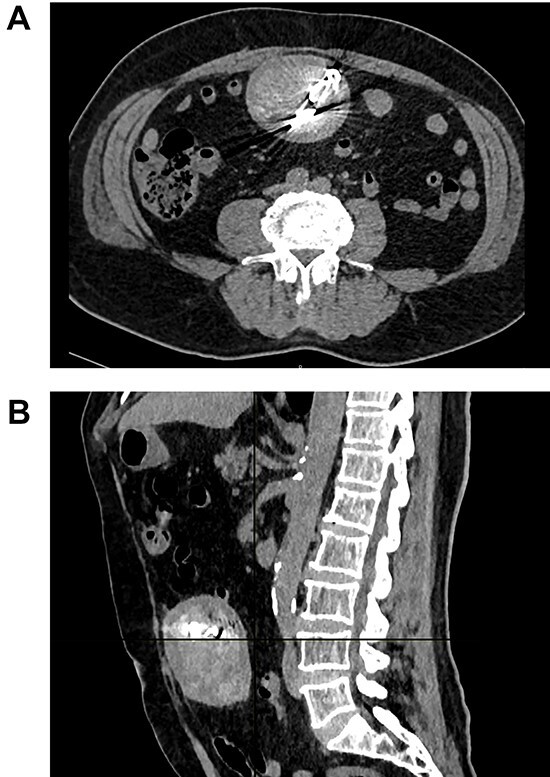
(**A**) CT the mass is seen in the abdomen with radiopaque material on its center. (**B**) CT the mass attached to the bowel.

Surprisingly, a well-encapsulated 7.2 × 6.3 × 8.6 cm spongiform mass with rounded edges and multiple radiodense linear calcifications on its center was discovered inside his abdomen (Fig. 1A and B).

With these findings and after obtaining consent, surgery was decided.

**Figure 2:**
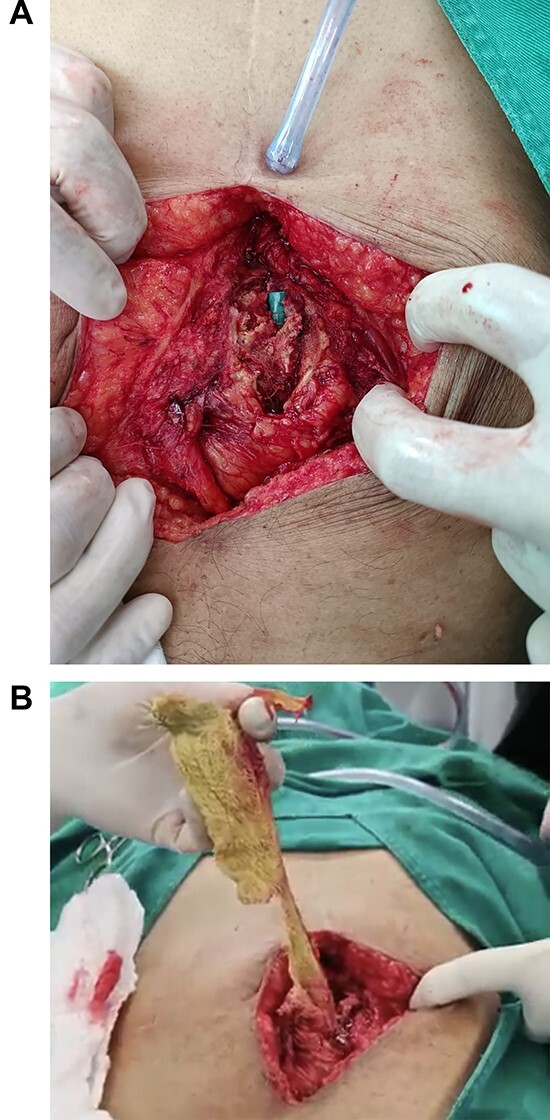
(**A**) the surgical cotton pad is seen in the abdomen. (**B**) Surgery, the pad is removed from the abdomen.

At laparotomy, the mass was found in the hypogastric region surrounded by the appendix, omentum and small intestine, along with an abscess of 150 cc. While removing the mass, the wall of the small bowel was extremely attached to it, and during this process, it was injured. Therefore, the compromised segment of the bowel was resected, and a side-to-side anastomosis was performed with a mechanical stapler. The enterotomy was closed in a two-layer fashion, a drain was placed, and the rest of the procedure was completed without complications (Fig. 2A and B).

Unfortunately, his postoperative course was troublesome, the drain became purulent and the patient continued to have a high fever even after surgery. Finally, on postoperative Day 5, intestinal fluid was noticed on the drain. A new laparotomy was necessary, and a small anastomosis leak was recognized within the highly inflamed bowel. Considering this, an ileostomy was required due to the patient’s condition.

After this event, the patient had a favorable outcome. He completed the antibiotic and learned to handle his ileostomy. He was discharged without any complications. On follow-ups, although he is psychologically affected and in a legal process against his previous physician, he is doing well.

Pathology reported retained surgical sponges surrounded by a fibrous capsule.

## DISCUSSION

During any surgical procedure, the primary determinant of patient safety and outcome is the skill and judgment of the surgeon and his team [1]. Regardless, making mistakes is and always will be normal human behavior [1, 2]. However, when these errors have significant consequences or occur in high-risk careers, such as surgery, they are of paramount importance [2]. Medical errors are often highly publicized, which attracts public attention and negatively affects surgeons [1].

However, because surgery is a dynamic specialty, it is prone to more errors, even with strict guidelines, newer tools, improved surgical safety protocols and minimally invasive techniques [1, 2]. Unfortunately, it has been demonstrated on multiple occasions that the majority (54%) of surgical errors can be avoided [2].

Since the first description by Wilson *et al*. in 1884, the term gossypiboma has been used to describe an iatrogenic mass caused by a surgical gauze or sponge accidentally left behind in a previous surgery [3]. Its incidence is uncertain, mainly due to staff fear of retaliation against them, so it is presumably underreported; however, it is believed to occur in 1 in 5500–10 000 surgeries [3, 4].

Surgical sponges are the most commonly forgotten object, and the most affected cavity is the abdomen (56%), followed by the thorax (11%) and pelvis (8%) [5]. However, this unfortunate event can occur in any surgery, including cholecystectomy, cesarean section or hysterectomy [3, 5]. In addition, several risk factors have been identified that increase the risk of this complication, such as open surgery, emergency surgery, unplanned change of operating room staff and a high body mass index [4]. In our case, the surgery was a prostatectomy performed 24 years ago.

Forgetting a surgical object can result in two types of responses, transudative, when the foreign body has a low inflammatory response, and exudative, when there is high antigenicity in the foreign body resulting in severe inflammation and abscess formation [3, 6]. In addition, if a foreign body is retained for a considerable period, it can lead to encapsulation, adhesions and calcifications [6], as it happened to our patient.

Clinical presentation is highly variable [3, 4]. Patients may present within days or even years; most will remain asymptomatic for a long time [5, 6]. However, they may present with obstruction, peritonitis, adhesions, abscesses or erosions [5, 7]. Diagnosis is usually made by imaging; plain radiographs may show a radiopaque marker if it is impregnated in the surgical gauze, and ultrasound may show a cystic mass with heterogeneous contents [6]. CT is the most commonly used imaging method; it can reveal surrounding organ involvement and aid in perioperative planning [7], as we did with our patient.

Gossypiboma can only be treated by surgery [3, 8]. However, it carries a high risk of reoperation, readmission, prolonged hospital stay, sepsis, fistulas and death [3–5]. All this without considering the costs (>$150 000 on average) for the patient and the hospital, as well as the medico-legal consequences for the surgeon and his team [6, 9].

In our case, the gossypiboma caused an acute abdomen that, despite our efforts, led to severe complications and long-term morbidity.

This complication is frequently referred to as a ‘never event’, as it can cause significant morbidity for the patient and repercussions for the medical team. There are several techniques to prevent this problem, including various checklists and safety protocols, all this to ensure an optimal environment within the operating room, as it is the collective responsibility of the entire team, not just the surgeon, to ensure the safety of every patient entering the operating room.

## CONCLUSIONS

Gossypiboma is a severe, preventable but devastating complication with dangerous medical and legal repercussions due to human error. These troublesome events can only be prevented through constant communication, adherence to counting protocols and a high level of awareness to provide the patient with the safety they expect and need.

## CONFLICT OF INTEREST STATEMENT

No conflict of interest to disclose.

## FUNDING

None.
